# Sensory-specific peripheral nerve pathology in a rat model of Fabry disease

**DOI:** 10.1016/j.ynpai.2021.100074

**Published:** 2021-09-02

**Authors:** Tyler B. Waltz, Anthony J. Burand, Katelyn E. Sadler, Cheryl L. Stucky

**Affiliations:** Department of Cell Biology, Neurobiology and Anatomy, Medical College of Wisconsin, Milwaukee, WI, USA

**Keywords:** FD, Fabry disease, Gb3, globotriaosylceramide, α-GAL A, α-galactosidase A, DRG, dorsal root ganglia, TEM, transmission electron microscopy, LM, Light microscopy, IB4, isolectin B4, LAMP1, lysosomal-associated membrane protein 1, Fabry disease, Neuropathic pain, Nerve pathology, Globotriaosylceramide, Lysosome

## Abstract

Fabry disease (FD) causes life-long pain, the mechanisms of which are unclear. Patients with FD have chronic pain that mirrors symptoms of other painful peripheral neuropathies. However, it is unclear what underlying damage occurs in FD peripheral nerves that may contribute to chronic pain. Here, we characterized myelinated and unmyelinated fiber pathology in peripheral nerves of a rat model of FD. Decreased nerve fiber density and increased nerve fiber pathology were noted in unmyelinated and myelinated fibers from FD rats; both observations were dependent on sampled nerve fiber modality and anatomical location. FD myelinated axons exhibited lipid accumulations that were determined to be the FD-associated lipid globotriaosylceramide (Gb3), and to a lesser extent lysosomes. These findings suggest that axonal Gb3 accumulation may drive peripheral neuron dysfunction and subsequent pain in FD.

## Highlights

• Fabry disease rats exhibit peripheral nerve pathology, specifically in sensory nerves.
• Fabry disease tibial nerves exhibit different pathology according to anatomical location.
• Fabry disease associated lipids and lysosomes accumulate in peripheral axons.

## 1 Introduction

Fabry disease (FD) is an X-linked lysosomal storage disorder that results in deficiency of the lysosomal hydrolase enzyme α-galactosidase A (α-GAL A) (Germain et al., 2015, Germain, 2010, El Dib et al., 2016). Most patients with FD experience various painful phenotypes, including stimulus-evoked pain, acute pain crises, and persistent spontaneous pain (Uceyler et al., 2014, Politei et al., 2016). Patients with FD are typically diagnosed with painful small fiber neuropathy due to functional assessment of peripheral nerves (Politei et al., 2016, Üçeyler et al., 2011, Dütsch et al., 2002, Marchettini et al., 2006). While treatment of FD with α-GAL A enzyme replacement therapy slows the progression of FD symptoms (Alegra et al., 2012), the prevalence and severity of chronic pain in patients with FD remains high (Politei et al., 2016). Thus, there is a desperate need to understand the mechanisms leading to FD peripheral neuropathy.

Sensory nerves from patients with FD exhibit pathology (Sima and Robertson, 1978, Kocen and Thomas, 1970, Kahn, 1973, Torvin Møller et al., 2009, Toyooka and Said, 1997), including abnormal myelinated and unmyelinated fiber density, lipid occlusions in various cell types, myelin pathology, and denervated glial cells. These pathologies may stem from α-GAL A enzyme deficiency in FD, which leads to accumulation of the lipid globotriaosylceramide (Gb3) in various tissues. Previous literature reports Gb3 accumulation and swelling of dorsal root ganglia (DRG) somata in patients (Politei et al., 2016, Godel et al., 2017) and FD rodent models (Miller et al., 2018, Hofmann et al., 2018). However, the extent of Gb3 accumulation or other pathologies in FD peripheral nerves remains unclear. FD rodent models are a powerful tool for characterization of nerve pathology. If animal models recapitulate human pathology, they could be utilized to advance the understanding of pain mechanisms for patients with FD (Burand and Stucky, 2021).

In this study, we examined nerve pathology in the FD rat. Since FD is caused by deficient breakdown of Gb3 in lysosomes, we then determined if peripheral nerve pathology correlated with accumulation of Gb3 or lysosomes in the axons. Insights from structural changes to the axons may indicate a mechanism responsible for the development of FD neuropathy.

## 2 Methods

### 2.1 Animal model and tissue collection

**Fig. 1:**
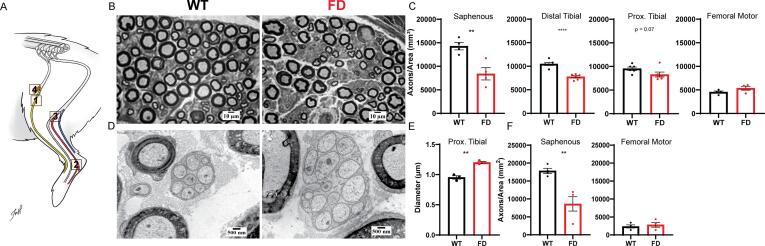
FD rat exhibits sensory-specific peripheral nerve pathology. (A) Anatomical diagram of rat hind limb illustrating location of the 1) saphenous nerve (sensory), 2) distal and 3) proximal tibial nerve (mixed sensory/motor), and 4) femoral motor branch. (B) Representative light microscopy (LM) images of myelinated fibers in the distal tibial nerve. (C) Quantification of myelinated fiber density in saphenous, distal tibial, proximal tibial, and femoral motor nerves. (D) Representative transmission electron microscopy (TEM) image of unmyelinated fibers in the proximal tibial nerve. (E) Quantification of unmyelinated axon diameter in proximal tibial nerve. (F) Quantification of unmyelinated fiber density in the saphenous and femoral motor nerves. Each dot represents the average value from one animal, n = 3–6 animals per genotype; unpaired Students *t*-test: * p < 0.05, ** p < 0.01, **** p < 0.0001.

The Fabry disease (FD) rat model (Miller et al., 2018) (Rat Genome Database symbol: Gla^em^^2Mcwi^ ) was compared to wild-type (WT) littermate controls. All rats included in this report were obtained from the existing FD rat colony at the Medical College of Wisconsin. All rats were male and between the ages of 35–60 weeks old, ages at which mechanical hypersensitivity behaviors are consistently displayed (Miller et al., 2018). Animals were anesthetized (4% isoflurane) and nerves were then harvested. 5 mm of nerve was taken from 4 different locations. The tibial nerve was taken 5 mm distal to the sciatic bifurcation in the mid-thigh region (proximal) and 10 mm above the calcaneus (distal) (Fig. 1A). The saphenous nerve and the femoral motor branch (Jesuraj et al., 2012, Moore et al., 2010) were taken at their bifurcation from the main trunk of the femoral nerve (Fig. 1A). Motor function of the femoral motor branch and lack of motor function of the saphenous was confirmed by 0.5 mA electrical stimulation of the intact nerve in anesthetized animals. Tissue processing is described below for each imaging technique. All protocols were in accordance with National Institutes of Health guidelines and were approved by the Institutional Animal Care and Use Committee at the Medical College of Wisconsin (Milwaukee, WI; protocol 383).

### 2.2 Light microscopy (LM) and transmission electron microscopy (TEM)

Nerve tissue was processed as done previously (Lewis et al., 2017). TEM imaging was completed on a subset of LM tissue sets. Briefly, nerves were fixed with 4% paraformaldehyde and 2% glutaraldehyde in sodium cocodylate buffer at 4 °C overnight, then postfixed with 1% osmium tetroxide for 2 h at room temperature. After dehydration in a graded methanol series, sections were infused with epon, and 0.5 μm sections were cut on a microtome for light microscopy analysis and stained with toluidine blue. For TEM, thin sections (70 nm) of post-osmium tetroxide treated nerve were stained with 25% uranyl acetate and counterstained with lead citrate, then imaged with TEM (Hitachi H-600) operating at 75KV excitation energy.

### 2.3 LM and TEM analysis

Myelinated fiber density and accumulation pathology was calculated manually with captured LM images using ImageJ software. Accumulation pathology in axons was classified as either displaying accumulation or not displaying accumulation. Axons with osmophilic accumulations were counted as positive if at least half of the axon space was covered in osmophilic substance. Remak bundle pathology was manually assessed on captured TEM images using ImageJ software. Total Remak bundle area and individual axon area and diameters were measured. These measurements were used to determine the number of axons per Remak bundle and the relative density of the Remak bundle (area of axons within Remak bundle/area of Remak bundle). The unmyelinated fiber density of the saphenous and femoral motor branch nerves were collected through live manual counting using TEM, as done in a previously described protocol (Orita et al., 2013). All analyses were conducted by a researcher blinded to genotype.

### 2.4 Immunofluorescence

Nerves were processed using a modified version of a previously described protocol (Megat et al., 2019). Nerves were flash-frozen in OCT with liquid nitrogen. Sections of nerves (20 μm) were mounted onto SuperFrost Plus slides (Thermo Fisher Scientific, 12–550-15) and fixed in ice-cold 10% formalin for 15 min, then dehydrated in an ethanol gradient (50, 70, and 100%; 5 min each). Nerve sections were stained using a modified version of a previously described protocol (Bartsch et al., 2019). Slides were permeabilized with 0.05 M Tris-buffered saline with 0.3% Triton-X (TBS/TX, 2.7% NaCl, pH 7.6) (Sigma-Aldrich) for 1 hr then washed. Nerve sections incubated in primary antibody solution (antibody info below; in TBS/TX + 10% normal serum) at 4 °C overnight and were washed. Secondary antibodies were placed into TBS/TX + 10% normal serum and slides incubated for 2 h at room temperature. Slides were subsequently washed, mounted with ProLong™ Gold Antifade Mountant (Thermo Fisher Scientific, P10144), and imaged using a confocal microscope (Leica TCS SP8). For a negative control, isotype or pre-absorption controls were utilized to determine specificity of primary antibody staining. Incubation of IB4 with 500 mM galactose was done to demonstrate lectin specificity (Miller et al., 2018); no significant IB4 staining was observed with 500 mM galactose.

Primary antibodies used included anti-NF200 (Sigma, N5389) at 1:1200, anti-LAMP1 (Abcam, Ab24170) at 1:400, and biotinylated isolectin B4 (IB4) (Vector, ZG0707) at 1:400. Secondary antibodies used were goat anti-mouse, AlexaFluor 588 (Invitrogen, A-11032) at 1:200, donkey anti-rabbit IgG, AlexaFluor 488 (Invitrogen, A-21206) at 1:800, and Streptavidin conjugated Cy™3 (Jackson, 016–160-084) at 1:1000.

### 2.5 Co-localization analysis

**Fig. 2:**
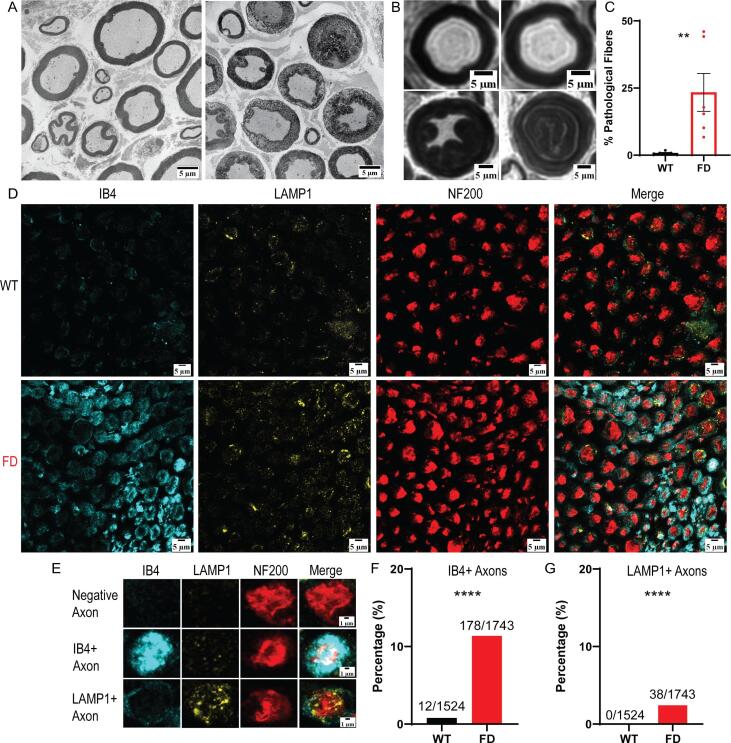
FD rat tibial nerve possesses extensive lipid accumulation in axons. (A) Representative TEM images of WT (left) and FD (right) osmophilic accumulation pathology. (B) Representative LM images of normal (top) and lipid accumulated axons (bottom). (C) Manual analysis of accumulation pathology. (D) Representative immunofluorescent images of wild-type (WT) and FD rat proximal tibial nerve. (E) Examples of negative, IB4+ (Gb3), and LAMP1+ axons taken from Fig. 2D. (F) Quantification of IB4+ (Gb3) and (G) LAMP1+ axons in FD compared to controls. Each dot represents the average value of one animal. Light microscopy, n = 6 animals per genotype, immunofluorescence, n = 3 animals per genotype, ** p < 0.01, **** p < 0.0001.

All microscope settings were kept consistent between images and genotypes to allow for quantification. To determine positive IB4 or LAMP1 axons, 30 NF200+ axons that were determined to be negative for IB4 and LAMP1 were manually assessed for baseline mean IB4 and LAMP1 fluorescence within a NF200 + axon. Any axon with LAMP1 or IB4 signal that was three standard deviations above this baseline mean were considered LAMP1 or IB4 positive; see Fig. 2E for examples. Co-localization of LAMP1 to NF200 and IB4 to NF200 was automatically collected using an ImageJ macro (https://github.com/tywaltz/Co-localization-analysis-in-nerve-cross-section). The observer was blinded to genotype during analysis.

### 2.6 Statistics

All data are presented as mean ± SEM. All analyses were done using GraphPad Prism version 9.1.0. Single comparisons were performed using Student's t test, and co-localization analysis was analyzed using Fisher's Exact Test.

## 3 Results

### 3.1 The FD rat exhibits sensory-specific peripheral nerve pathology

Morphological abnormalities are observed in peripheral nerves of patients with FD (Kocen and Thomas, 1970, Kahn, 1973, Torvin Møller et al., 2009, Toyooka and Said, 1997). To determine if there are morphological differences between FD and wild-type (WT) rats, we sampled various peripheral nerves (Fig. 1A), including the saphenous nerve (sensory), the tibial nerve (mixed sensory/motor) at proximal and distal locations, and the femoral motor branch (Jesuraj et al., 2012, Moore et al., 2010). A significant decrease in myelinated fiber density was observed in the saphenous (sensory) and distal tibial nerves (mixed sensory/motor) of FD rats (Fig. 1B). This finding is consistent with decreased intra‐epidermal nerve fiber density (IENFD) in patients with FD (Politei et al., 2016, Üçeyler et al., 2011, Schiffmann et al., 2006). No changes in myelinated fiber density were noted in FD proximal tibial (mixed sensory/motor) or femoral motor branches.

Next, unmyelinated fiber pathology was characterized in the tibial nerve (mixed sensory/motor). Significantly larger unmyelinated axon diameters (Fig. 1E) were observed in the proximal (Fig. 1D), but not distal, portion of FD tibial nerve. These observations are similar to the increased somata diameter observed in the DRG of FD rats (Miller et al., 2018) and patients (Gadoth and Sandbank, 1983, Godel et al., 2018). The density of unmyelinated fibers was then calculated in the saphenous (sensory) and femoral motor branch nerves to determine if FD preferentially decreased sensory nerve unmyelinated fiber density. We found decreased unmyelinated fiber density in the saphenous (sensory) but not femoral motor branch of FD nerves (Fig. 1F).

### 3.2 Osmophilic accumulations comprised of Gb3, and to a lesser extent LAMP1 + lysosomes, were observed in the proximal tibial nerve of the FD rat

Upon closer examination of the peripheral nerves, we found significant osmophilic accumulations in myelinated axons of the proximal tibial nerve (Fig. 2A-B). This finding was not seen in unmyelinated or myelinated fibers of the other peripheral nerves, including the distal tibial nerve. Upon quantification, an average of 23% of FD rat axons displayed a partial or full osmophilic accumulation within myelinated axon tracts (Fig. 2C).

Osmium tetroxide preserves a broad spectrum of lipid species in histological analysis (Wigglesworth, 1975, Belazi et al., 2009), thus these osmophilic accumulations likely represent accumulation of lipids. Based on the molecular basis of FD, it is likely that these lipid accumulations represent Gb3 and/or lysosomal accumulation. Therefore, we conducted immunofluorescent staining using antibodies against isolectin-B4 (IB4) and lysosomal-associated membrane protein 1 (LAMP1) to determine if these accumulations are Gb3 (Miller et al., 2018, Kirkeby and Moe, 2001) or LAMP1 + lysosomes (Eskelinen, 2006) respectively (Fig. 2D-E). Neurofilament 200 (NF200) was used as a background myelinated axon stain (Friede and Samorajski, 1970). Around 11% of FD myelinated axons had significant axonal accumulation of Gb3 (Fig. 2F). LAMP1+ lysosomal accumulation was observed in approximately 2% of FD myelinated axons and 0/1524 wildtype axons surveyed (Fig. 2G). Therefore, the significant osmophilic accumulations observed in FD myelinated axon tracts are likely accumulations of the lipid Gb3, and to a lesser extent, LAMP1+ lysosomes.

## 4 Discussion

This study characterized peripheral nerve pathology in the FD rat. We found that FD sensory nerves had observable pathology, whereas motor nerve fibers had no observable pathology. This finding aligns with patient sequelae, as patients with FD experience chronic pain and altered somatosensation (Uceyler et al., 2014) and only subtle differences in motor function (Cocozza et al., 2018). The loss of sensory, but not motor nerve fibers could be attributed to the relatively high blood-nerve barrier permeability of the DRG that is further permeabilized during nerve injury (Sadler et al., 2019, Abram et al., 2006, Reinhold and Rittner, 2020, Devor, 1999). Various factors in the blood, such as FD-associated lipids (Üçeyler et al., 2018) or inflammatory mediators (Üçeyler et al., 2019, De Francesco et al., 2013), could readily access FD DRG, influencing FD peripheral nerve pathology (Choi et al., 2015, Shamash et al., 2002, Leung and Cahill, 2010). Additional studies will be required to understand the selective susceptibility of sensory nerves to FD-related damage.

We also found distinct differences in FD nerve pathology depending on the anatomical location of tibial nerve biopsy (Fig. 1A). Distal tibial nerves in the FD rat exhibited decreased fiber density similar to observations made in the skin (Üçeyler et al., 2011, Schiffmann et al., 2006) and the distal nerves of patients with FD (Sima and Robertson, 1978, Kocen and Thomas, 1970, Kahn, 1973, Torvin Møller et al., 2009). However, the proximal tibial nerve fiber density was similar between FD and WT control rats, but lipid accumulations and axonal swelling were observed in proximal myelinated and unmyelinated FD axons respectively. These proximal nerve pathologies may precipitate the dying back of distal fibers. For example, the subset of axons that exhibited accumulation proximally may be the same axon tracts that are retracting distally. However, future studies are needed to investigate this hypothesis.

To our knowledge, this is the first study that has quantified the extent of Gb3 and lysosome accumulation in peripheral axons of a preclinical FD model. The findings in this study suggest that the lipid accumulations observed in myelinated axons of patients with FD and the FD rat model are likely comprised of Gb3. LAMP1+ lysosomal accumulation was also observed in a small subset of FD myelinated axons. While lysosomes travel through axon tracts to maintain cellular health in the nerve terminals (Becker et al., 1960, Ferguson, 2018), they are not found in high concentrations within axon tracts in healthy states. Despite the low number of FD myelinated axons that exhibited lysosomal accumulation (around 2%), this was significantly higher than the accumulation observed in WT axons, and therefore, may suggest that organelle trafficking in FD axons is defective.

The pathological findings observed in this study could help explain functional differences seen in microneurography studies completed in patients with FD (Torvin Møller et al., 2009, Sheth and Swick, 1980, Üçeyler et al., 2013, Valeriani, 2004). Patients with FD exhibit abnormal myelinated Aδ fiber conduction (Üçeyler et al., 2013). Around 25% of FD rat myelinated axons exhibited massive lipid accumulation, representing a potential mechanism for the myelinated Aδ fiber dysfunction seen in patients with FD. C-fiber dysfunction is also observed in patients with FD, including changes of pain thresholds to pinprick, heat, and cold stimuli (Torvin Møller et al., 2009, Valeriani, 2004). Here, we showed increased unmyelinated axon diameters and sensory-specific loss of unmyelinated fibers. This finding suggests that the C-fibers of FD peripheral nerves could have altered nerve conduction (Costa et al., 2018), which may contribute to FD-associated pain. Our future studies will test this hypothesis.

From the immunofluorescent images (Fig. 2D), it is likely that Gb3 accumulates in both myelinated nerve fibers and other peripheral cell types. While FD pain is considered neuropathic in nature, recent evidence suggests FD pain may have an inflammatory component (Üçeyler et al., 2019, Sommer et al., 2018). The FD rat model has increased immune cell levels in the skin (Miller et al., 2019), so there may also be IB4+ macrophage infiltration occurring at the level of the peripheral nerve that accounts for the IB4 staining not co-localized with NF200 (Wright et al., 1983). In addition, accumulation of Gb3 in non-neuronal cells, such as Schwann cells, could account for the IB4 staining that is not co-localized with NF200+ myelinated axons. Pathology in these cell types may also contribute to nerve dysfunction and pain in FD (Wei et al., 2019, Ji et al., 2016).

The findings of this study further support the use of the preclinical FD rat model to predict mechanisms underlying peripheral neuropathy and pain in patients with FD (Miller et al., 2018). Our study showed that FD rat peripheral nerves exhibited axon enlargement and axon degeneration. These pathologies are also seen in other painful peripheral neuropathies, such as chemotherapy-induced and diabetic peripheral neuropathy (Fukuda et al., 2017, Calcutt, 2020). Therefore, FD may share mechanistic similarities to other painful peripheral neuropathies. However, our finding of lipid accumulation in FD nerves is unique in the context of other painful peripheral neuropathies. Interestingly, the phenotype of lipid accumulation and axon enlargement bears similarity to certain neurodegenerative disorders (Farmer et al., 2020, Adalbert and Coleman, 2013). Recent studies support a mechanistic link between glial cell lipid accumulation, dysregulation of lipid metabolism in neurons, and the neuropathology seen in Alzheimer’s disease (Farmer et al., 2020). Defects in microtubule-associated proteins, such as Tau, lead to axon swelling (Costa et al., 2018), axon degeneration (Saxena and Caroni, 2007), and diminished axonal transport of organelles (Meyer et al., 1995) which can cause protein accumulation. While the current literature has not fully investigated how α-GAL A deficiency influences microtubule function, expression of certain microtubule-associated proteins is modified by α-GAL A deficiency (Chévrier et al., 2010, Nelson et al., 2014). Therefore, further studies investigating the mechanisms of FD peripheral neuropathy could yield important findings for other neurological disorders.

## CRediT authorship contribution statement

**Tyler B. Waltz:** Conceptualization, Methodology, Investigation, Formal analysis, Visualization, Writing – original draft. **Anthony J. Burand:** Conceptualization, Formal analysis, Writing - review & editing. **Katelyn E. Sadler:** Conceptualization, Visualization, Writing - review & editing. **Cheryl L. Stucky:** Conceptualization, Writing - review & editing, Supervision, Funding acquisition.

## Declaration of Competing Interest

The authors declare that they have no known competing financial interests or personal relationships that could have appeared to influence the work reported in this paper.
